# Low Doses of Sucralose Alter Fecal Microbiota in High-Fat Diet-Induced Obese Rats

**DOI:** 10.3389/fnut.2021.787055

**Published:** 2021-12-28

**Authors:** Minchun Zhang, Jie Chen, Minglan Yang, Cheng Qian, Yu Liu, Yicheng Qi, Rilu Feng, Mei Yang, Wei Liu, Jing Ma

**Affiliations:** Department of Endocrinology and Metabolism, Renji Hospital, School of Medicine, Shanghai Jiao Tong University, Shanghai, China

**Keywords:** artificial sweeteners, sucralose, fecal microbiota, obesity, 16S ribosomal RNA gene analysis

## Abstract

Artificial sweeteners (AS) have been widely used as sugar substitutes to reduce calorie intake. However, it was reported that high doses of AS induced glucose intolerance via modulating gut microbiota. The objective of this study was to investigate the effects of lower doses of sucralose on fecal microbiota in obesity. Eight weeks after high-fat diet (HFD), the male Sprague Dawley rats were randomly divided into four groups (6 in each group) and administrated by a daily gavage of 2 ml normal saline (CON), 0.54 mM sucralose (N054), 0.78 mM sucralose (N078), and 324 mM sucrose (S324), respectively. After 4 weeks, fecal samples were obtained and analyzed by 16S ribosomal RNA gene sequencing. The richness and diversity of fecal microbiota were not changed by sucralose or sucrose. Both 0.54 mM (0.43 mg) and 0.78 mM (0.62 mg) sucralose tended to reduce the beneficial bacteria, *Lactobacillaceae* and *Akkermansiaceae*. The relative abundance of family *Acidaminoccaceae* and its genus *Phascolarctobacteriam* were increased after 0.54 mM sucralose. In functional prediction, 0.54 mM sucralose increased profiles of carbohydrate metabolism, whereas 0.78 mM sucralose enhanced those of amino acid metabolism. The lower doses of sucralose might alter the compositions of fecal microbiota. The effects of sucralose in different dosages should be considered in the future study.

## Introduction

Obesity has emerged as a major public health challenge affecting over 650 million adults worldwide. It increases the risks of type 2 diabetes, cardiovascular diseases, and even certain cancers (1). Table sugars contribute to the weight gain and thereby risks for metabolic disorders (2, 3). Therefore, artificial sweeteners (AS) are widely used as sugar substitutes to provide intensive sweet taste without extra calorie.

The US Food and Drug Administration (FDA) provided the acceptable daily intake (ADI) levels of 6 kinds of AS including saccharin, aspartame, acesulfame potassium (Ace-K), sucralose, neotame, and advantame (4). However, the effects of AS on glucose homeostasis remain controversial. Some studies demonstrated the benefits of AS exposure (5), whereas others showed that AS were associated with the incidence of obesity and type 2 diabetes (6–8).

The plausible mechanisms underlying the metabolic effects of AS are not fully understood. Given that most AS pass through the gastrointestinal tract without being absorbed or digested, they may directly alter the gut microbiota which plays crucial roles in the pathogenesis of metabolic diseases (9, 10). Suez et al. reported that saccharin in ADI dose (5 mg/kg body weight) induced glucose intolerance by modulating gut microbiota in mice and healthy subjects (11). The transplantation of saccharin-exposed feces induced glucose intolerance in germ-free mice (11). Another, it was indicated that administration of sucralose at dosages of 1.1–11 mg/kg reduced beneficial fecal bacteria and elevated fecal pH, intestinal p-glycoprotein, and cytochrome p-450 in rats (12). It should be noticed that the doses of AS in most studies were far beyond levels of daily consumption.

Sucralose is derived from sucrose with replacement of three hydrogen–oxygen groups by three chlorine atoms. In this process, the sweetness of sucralose is dramatically intensified to about 600 times of sucrose (13). About 85% of sucralose is excreted without being absorbed or digested in the gastrointestinal tract (13). Previous studies showed that a single dose of sucralose had no effects on blood glucose in health subjects (14) and patients with type 2 diabetes (15). However, it has been reported that sucralose exerted strong bacteriostatic effects *in vitro* and altered the structures of microbial communities in normal rodents (16). It remains unclear whether sucralose particularly in low doses can modulate the gut microbiota compared with natural sugars. We therefore aimed to evaluate the potential effects of different concentrations of sucralose and sucrose on fecal microbiota in high-fat diet (HFD)-induced obese rats.

## Materials and Methods

### Animals

Male Sprague Dawley (SD) rats (4 weeks old) were fed with sterile food and water under specific pathogen-free (SPF) conditions with 12-h dark–light cycle, controlled temperature (20–23°C), and settled humidity (40–60%) (Laboratory Animal Resources, Chinese Academy of Sciences). After adapting to the environment for 1 week, the rats were fed with an *ad libitum* HFD (45% fat) or normal chow diet (NCD, 10% fat) correspondingly for 8 weeks. Rats on HFD weighed 20% more than those on the NCD group were considered as obesity. The protocol of this study was approved by the Institutional Animal Care and Use Committee of Shanghai Laboratory Animal Center, Chinese Academy of Sciences on January 8, 2018.

### Treatment

The 24 obese rats were randomly divided into 4 groups (6 in each group): normal saline (control group, CON), 0.54 mM sucralose (N054, Sigma-Aldrich, MO, USA), 0.78 mM sucralose (N078), and 324 mM sucrose (S324, Sigma-Aldrich, MO, USA). Rats were intragastric administrated with 2 ml certain solution at a fixed time every day for 4 weeks (17). The doses translated to human were 0.11 mg/kg (N054), 0.16 mg/kg (N078), and 56.20 mg/kg (S324) according to the body surface area (18).

### Fecal Sample Collection

At the end of treatment, fecal samples were collected after 12-h fasting. Each rat was hold in hands and received abdominal massage until fresh pellets were collected in a 1.5-ml sterile freezing tube. The tubes were placed immediately in liquid nitrogen and moved to −80°C refrigerator.

### DNA Extraction, PCR Amplification, and 16S rRNA Gene Sequencing

DNA extraction, PCR amplification, and 16S rRNA sequencing were performed as described in previous study (19). In short, total genomic DNAs of stool samples were extracted using the EZNA soil DNA Kit (Omega Bio-Tek, Norcross, GA, USA). Genes of the 16S rRNA V3–V4 regions were amplified by specific 338F and 806R primers with thermocycler polymerase chain reaction (PCR) system (GeneAmp 9700, ABI, USA). The extracted and purified amplicons were sequenced using Illumina MiSeq platform (Illumina, San Diego, USA).

### Statistical Analyses

All data were included in the analysis. Bioinformatic analyses were performed by the Majorbio I-Sanger Cloud Platform (https://cloud.majorbio.com/) and SPSS Statistics v.23 software (IBM). Alpha diversity indices were applied to analyze the richness and diversity of samples, including Sobs, ACE, Chao1, Shannon, and Simpson. Unsupervised principal coordinates analysis (PCoA) and supervised partial least squares-discriminant analysis (PLS-DA) were performed to explore the similarities or dissimilarities of each sample. Permutational multivariate ANOVA (PERMANOVA) was calculated on the base of Bray–Curtis.

Differences in the relative abundance of taxa among groups were analyzed using the Kruskal–Wallis rank sum test with Tukey–Kramer *post-hoc* analysis. Correlation network according to Spearman's correlation analysis was used to determine the interactions of bacterial community. The linear discriminant analysis (LDA) effect size (LEfSe) algorithm differentiated microbial features for biomarker discovery. Only taxa with absolute LDA (log10) scores >2.0 and a p value of 0.05 were presented in this study. Metabolic functions were predicted using Phylogenetic Investigation of Communities by Reconstruction of Unobserved States (PICRUSt).

## Results

### Characteristics of Bacterial Diversity and Clustering

**Figure 1:**
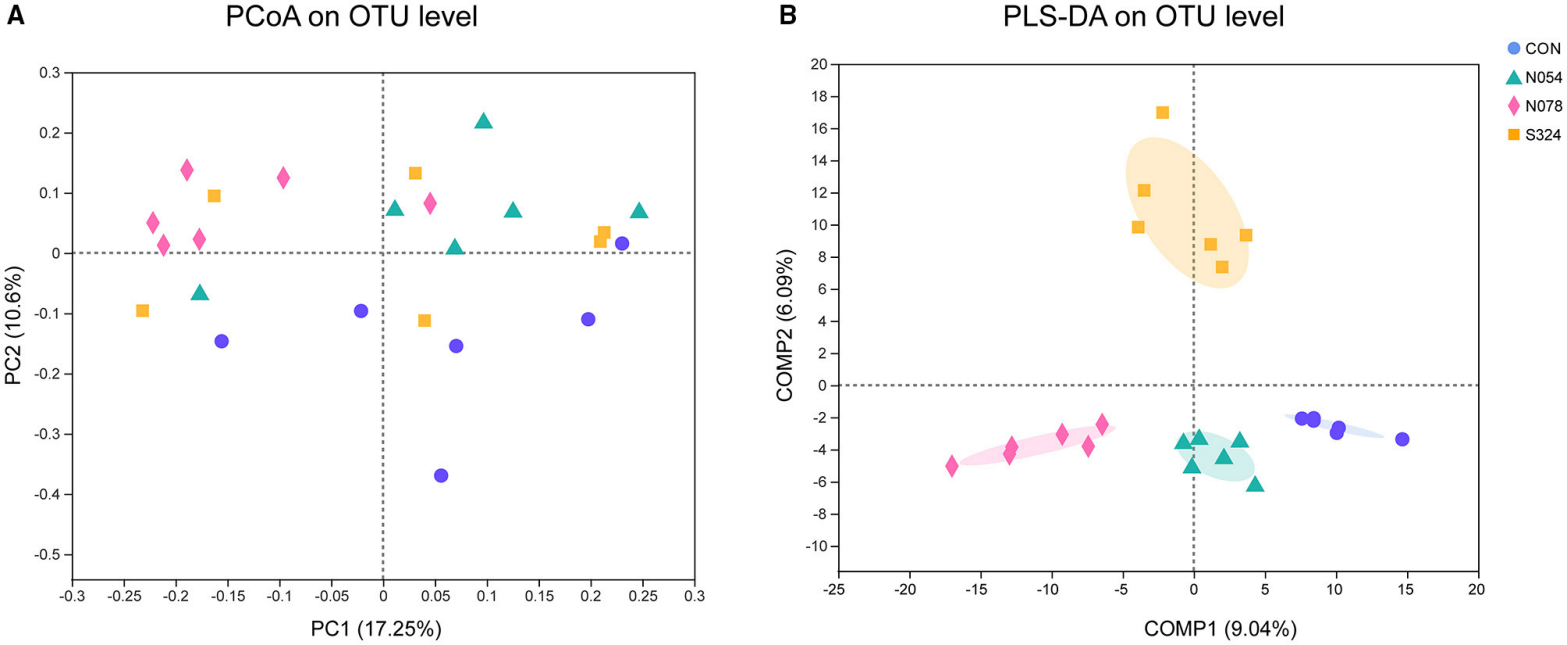
Beta diversity analysis in four groups with unsupervised and classification methods. **(A)** Principal coordinates analysis (PCoA). Bray–Curtis distances and permutational multivariate analysis of variance (PERMANOVA) were performed. *p* = 0.001 and *p* adjust = 0.001. **(B)** Partial least squares discriminant analysis (PLS-DA). Each sample was represented by a dot (*n* = 6). CON, control group; N054, 0.54 mM sucralose; N078, 0.78 mM sucralose; S324, 324 mM sucrose.

In the analysis of alpha diversity, neither sucralose nor sucrose altered the community richness (Sobs, ACE, Chao1 index) or diversity (Shannon, Simpson index) of fecal microbiota (Supplementary Table S1). The PCoA plot revealed that most samples treated by 0.78 mM sucralose clustered in a distinct group compared with CON, N054, and S324 groups (PERMANOVA, *p* = 0.001 and *p* adjust = 0.001). It was also confirmed by the supervised PLS-DA on OTU level. Each of the four groups showed a specific cluster (COMP1 9.04% and COMP2 6.09%), suggesting that they had different bacterial structures (Figure 1). The results of weighted unifrac and unweighted unifrac were similar to PCoA based on Bray–Curtis (Supplementary Figures S1A,S1B).

### Alterations of Core Microbial Composition Induced by Sucralose and Sucrose

**Figure 2:**
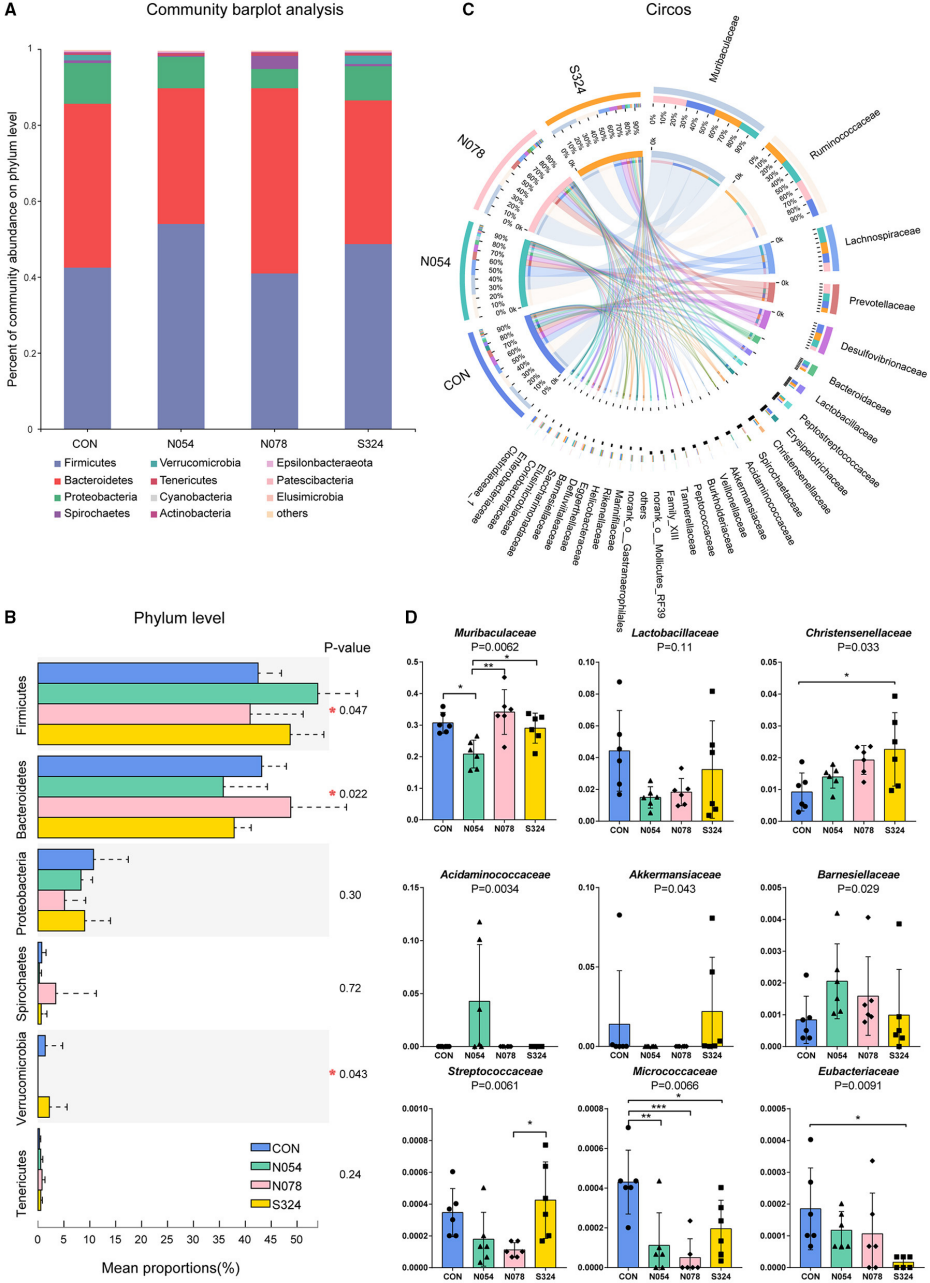
Main bacterial communities of different taxonomies. **(A)** Community bar plot of the domain phyla. **(B)** Relative abundant of the domain phyla of four groups. **(C)** Circos plot showing the relationship between microbial families and samples. **(D)** Relative abundance of core bacterial families. Kruskal–Wallis rank sum test with Tukey–Kramer *post-hoc* analysis was performed (*n* = 6). Mean ± standard error. *p < 0.05, **p ≤ 0.01, ***p ≤ 0.001.

On phylum level, 0.54 mM sucralose increased the relative abundance of *Firmicutes* but decreased that of *Bacteroidetes*. 0.78 mM sucralose decreased the relative abundance of *Firmicutes* but increased that of *Bacteroidetes* (Figures 2A,B). The ratio of *Firmicutes* to *Bacteroidetes* in N054 was higher than that in N078 (Supplementary Figure S2). No differences were detected in the ratio of *Bacteroidetes* to *Proteobacteria*. Notably, both 0.54 and 0.78 mM sucralose reduced the relative abundance of *Verrucomicrobia*.

**Figure 3:**
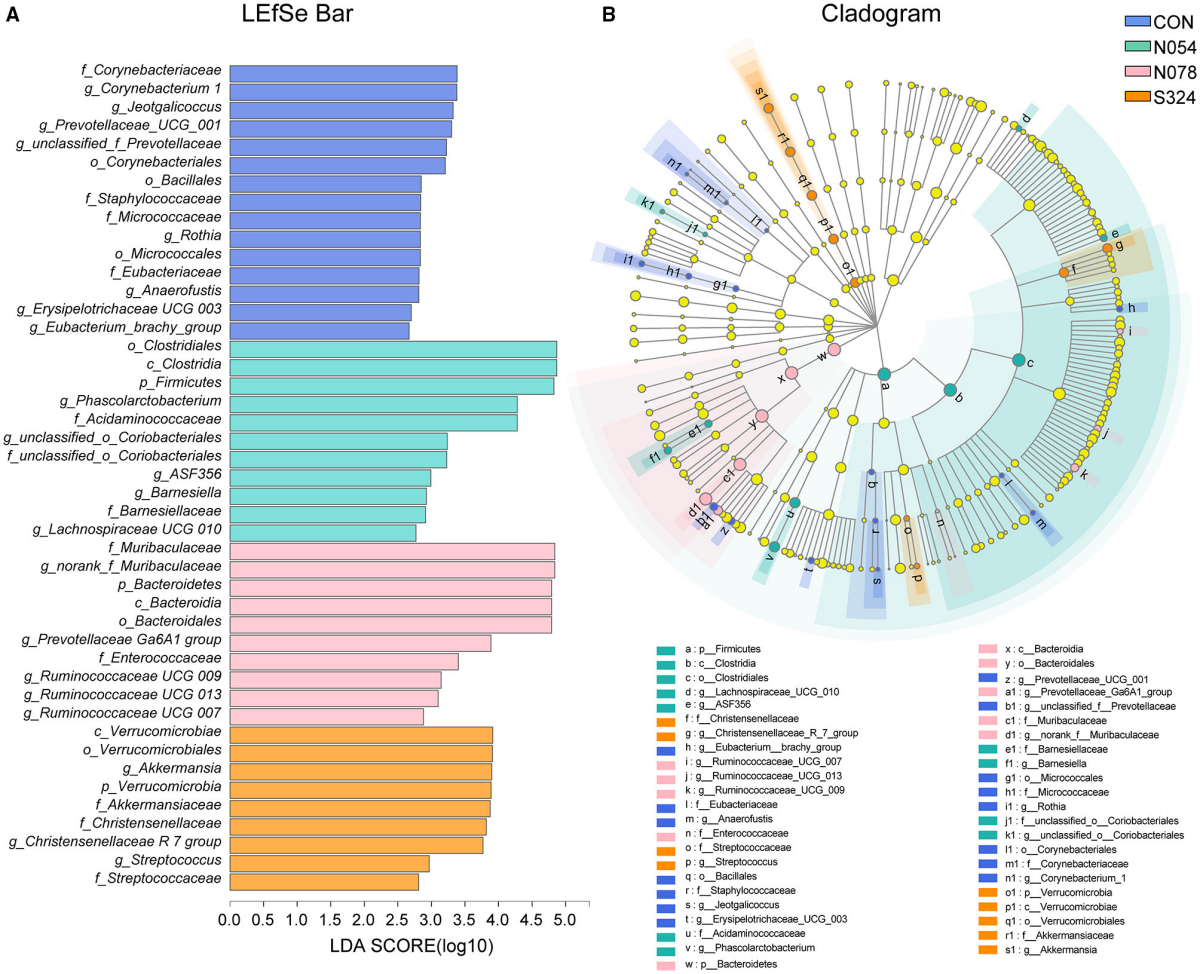
LDA effect size (LEfSe) analysis based on genus level among four groups. **(A)** LEfSe bar plot demonstrating the significant bacterial differences. **(B)** Cladogram indicating the phylogenetic distribution of fecal microbiota with phyla in the outermost and genera in the innermost ring. Multiple comparison strategy was all-against-all (*n* = 6). Only LDA score >2.0 is shown.

To describe the alterations of bacterial communities, the relative abundance of families was detected (Figures 2C,D). The beneficial bacteria, *Lactobacillaceae* and *Akkermansiaceae*, tended to be lower in both 0.54 and 0.78 mM sucralose, compared with control and sucrose groups (Figure 2D). These concentrations of sucralose increased the relative abundance of *Barnesiellaceae*, whereas they decreased that of *Streptococcaceae*. Sucralose and sucrose consistently upregulated *Christensenellaceae* and downregulated *Micrococcaceae* and *Eubacteriaceae*. 0.54 mM sucralose significantly reduced the relative abundance of *Muribaculaceae* but increased that of *Acidaminococcaceae*. LEfSe analysis showed that genus *Phascolarctobacterium*, belonged to the family *Acidaminococcaceae*, was enriched in N054 group. Family *Muribaculaceae* (S24-7) was enriched in N078 group (Figure 3) and it was negatively correlated with the change of body weight (Supplementary Figure S3). The family *Akkermansiaceae* and genus *Akkermansia* of *Verrucomicrobia* phylum were significantly enriched in S324 group (Figure 3).

In the network graph of interacting families in N054 group (Supplementary Figure S4), *Akkermansiaceae* was positively correlated with *Christensenellaceae, Barnesiellaceae, Veillonellaceae*, and *norank Gastranaerophilales* and it had a negative correlation with *Acidaminococcaceae*. The most abundant family *Muribaculaceae* had a positive interaction with *Bifidobacteriaceae* and negative interactions with *Deferribacteraceae* and *Burkholderiaceae*.

### Effects of Predicted Metabolic Functions of Fecal Microbiota

**Figure 4:**
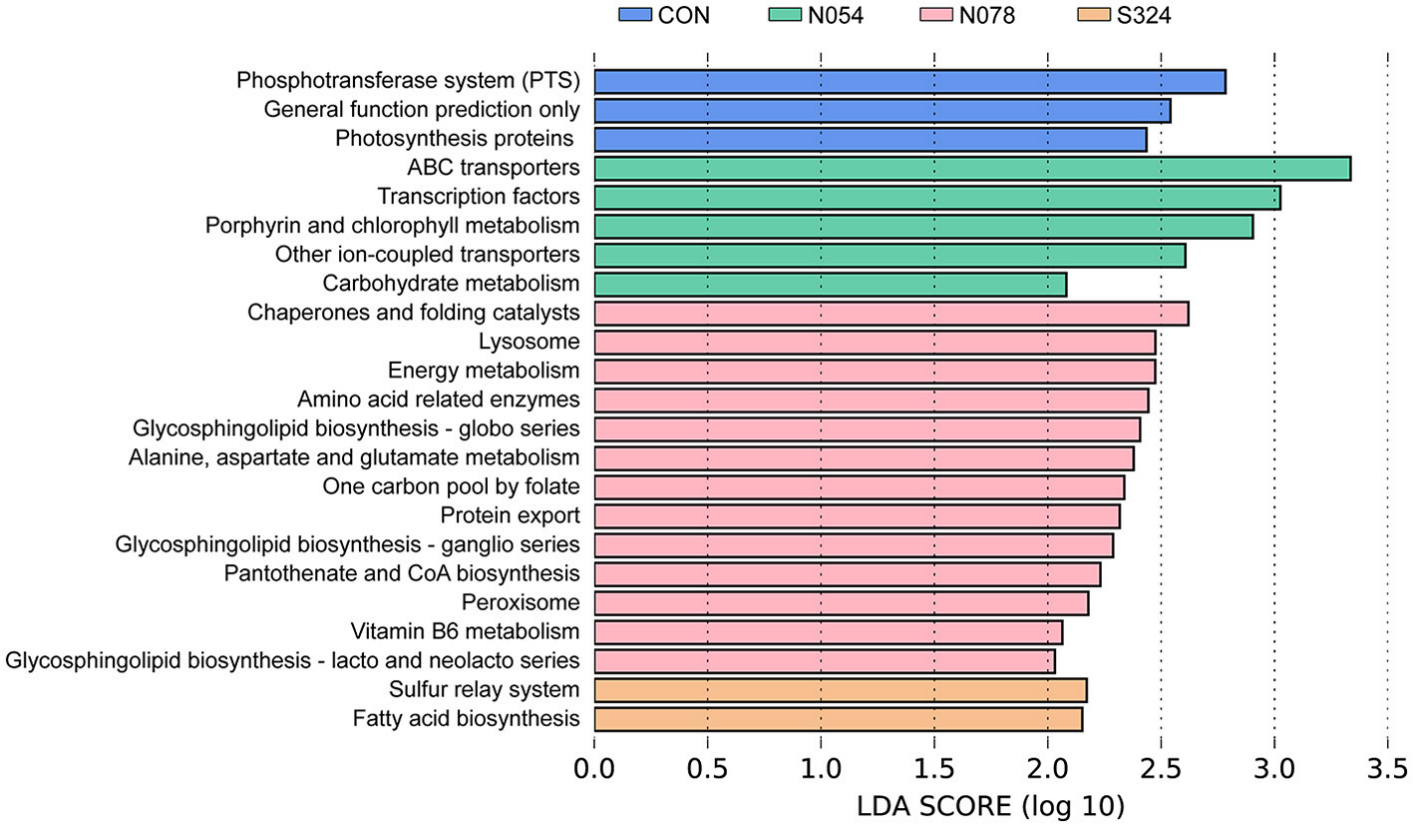
LEfSe analysis on predictive functions of KEGG level 3 identified *via* PICRUSt. A Log LDA >2.0 was considered as significant difference. KEGG, Kyoto Encyclopedia of Genes and Genomes; PICRUSt, Phylogenetic Investigation of Communities by Reconstruction of Unobserved States.

PICRUSt and LEfSe were used to determine the changes in predicted functional composition (Figure 4). At KEGG level 3, ATP-binding cassette (ABC) transporters and the carbohydrate metabolism were enhanced by N054 group. The exposure of 0.78 mM sucralose increased the functional profiles related to metabolism including amino acid-related enzymes, energy metabolism, alanine, aspartate and glutamate metabolism, pantothenate and CoA biosynthesis, and vitamin B6 metabolism. The biosynthesis of fatty acid was related to the 324 mM sucrose intervention.

## Discussion

In this study, we demonstrated 4-week low doses of sucralose (0.54 and 0.78 mM) altered the compositions and metabolic functions of fecal microbiota in obese rats. The richness and diversity of fecal microbiota were not changed by the sucralose and sucrose. Previous *in vitro* studies found that sucralose exerted bacteriostatic effects in a dose-dependent manner *via* inhibiting the invertase and sucrose permease of bacteria (16). However, sucralose did not reduce the overall richness and diversity of intestinal bacteria *in vivo* which was consistent with our results (16). It was probably due to the wide variety of microorganisms and their complex interactions with each other (20).

Beta diversity was used to explore the differences and similarities of microbial compositions among samples. Few studies investigated the impacts of AS on beta diversity. There was a study found that neotame changed the beta diversity after 4-week intervention on CD-1 mice (21). We presented that 0.54 mM (~0.43 mg) and 0.78 mM (~0.62 mg) sucralose groups had different clusters. It indicates that even the low doses of sucralose significantly altered the structures of fecal microbiota.

*Firmicutes* and *Bacteroidetes* were the two most abundant phyla, accounting for over 90% of the gut microbiota (22). We observed that 0.54 mM sucralose increased the relative abundance of *Firmicutes* and decreased that of *Bacteroidetes*, whereas 0.78 mM sucralose exerted the opposite effects. Notably, it was reported that sucralose did not alter the levels of *Firmicutes* nor *Bacteroidetes* in human (780 mg/d, 7 days) (23) nor mice studies (1.5 and 15 mg/kg body weight, 8 weeks) (24). Nevertheless, when sucralose was consumed with HFD simultaneously, there were obvious changes in *Firmicutes* and *Bacteroidetes*. A recent study also highlighted the intake of sucralose with carbohydrate impaired insulin sensitivity and glucose metabolism (25). Given the widely use of AS in obese patients, the interaction between AS and HFD warrants further study.

We presented that sucralose had no effects on phylum *Proteobacteria* level in HFD rats, which was consistent with the previous study (16). It was reported that *Proteobacteria* was elevated after the commercial sucralose (Splenda) dosage in a Crohn's disease model (SAMP mice) and the related control (AKR/J mice) (26). In fact, the higher level of *Proteobacteria* was closely related to inflammation, and it increased in the models of immune system dysfunction (27). Therefore, the effect of sucralose on *Proteobacteria* needs to be further clarified.

Our results highlighted that both 0.54 and 0.78 mM sucralose tended to reduce the relative abundance of beneficial bacteria *Lactobacillaceae* and *Akkermansiaceae*, which could improve metabolic symptoms via various mechanisms. Notably, *Lactobacillus* were reduced by 39.1% after a 12-week intervention of Splenda in healthy rats (12). The reduction of *Lactobacillus* was also confirmed in acesulfame potassium-treated mice (28). *Akkermansia Muciniphila*, a mucin-degrading bacterium, was lower in human or animal models with obesity and type 2 diabetes (29). Bian et al. observed that the abundance of *Akkermansia* was not changed during 3-month supplementation of sucralose but it was increased after further 3-month consumption in healthy mice (30). In our study, a 4-week administration with sucralose reduced *Akkermansiaceae* of obese rats. The underlying mechanisms are poorly understood. It is noteworthy that the osmolarities of solutions were different, which could mediate gastrointestinal motility directly and further affect community composition of intestinal flora (31). *Akkermansia* was decreased in anorexia nervosa after refeeding, the latter being accompanied by normal bowel movements (32). Although in our previous study, sucralose (0.4 mM nor 4 mM) had no effect on gastric emptying rate in healthy humans (14), its potential effects on gut microbiota are still unclear. Gastrointestinal tract transit times need to be investigated in future research.

In this study, sucralose at the dose of 0.78 mM increased family *Muribaculaceae* (S24-7), which was enriched in obesity-resistant mice (33). Sucralose and sucrose consistently upregulated *Christensenellaceae*, and the latter was inversely related to host body mass index (BMI) in several studies (34). Bian et al. showed the similar change after 6-month supplementation with sucralose into the drinking water (30). Another, *Eubacteriaceae, Barnesiellaceae, Streptococcaceae*, and *Micrococcaceae* were not closely correlated with metabolic disorder at present.

We demonstrated that the family *Acidaminoccaceae* was negatively associated with *Akkermansiaceae* in the network analysis. The genus *Phascolarctobacteriam*, belonging to family *Acidaminoccaceae*, was strongly correlated with metabolic dysfunction including weight gain and glucose intolerance (35). We found that *Phascolarctobacteriam* was enriched in the 0.54 mM sucralose group. *Phascolarctobacterium* could ferment carbohydrate and produced short-chain fatty acids such as acetate and propionate (36). It was consistent with our functional prediction that carbohydrate metabolism was enhanced in 0.54 mM sucralose dosage.

In accordance with the changes in bacterial compositions, we provided evidence that sucralose in doses of 0.54 and 0.78 mM changed functional profiles of fecal microbiota related to the metabolism of carbohydrates and amino acids. Gut microbial metabolite from daily diet was linking to the development of obesity and insulin resistance (37). Sucralose was previously showed to alter the metabolism of some amino acids and their derivatives (30). Additionally, Suez et al. (11) reported that the consumption of saccharin in ADI dosage increased the pathway genes related to glycosaminoglycan and other glycan. We presented that the dose of sucralose was an important factor to gut microbiota. Particularly, 0.54 mM sucralose (~2.2% of FDA ADI dosage) enhanced the ABC transporters and carbohydrate metabolism, whereas the exposure of 0.78 mM sucralose (~3.2% of ADI dosage) was more related to the amino acid metabolism. We previously indicated that 0.78 mM instead of 0.54 mM sucralose lowered the blood glucose level of HFD-induced obese rats (17). It should be noticed that the different effects of these sucralose dosages on gut microbiota might be partly responsible for the distinct energy metabolism. Thus, AS might have complex effects on fecal microbiota, taste receptors, and gut hormone secretion.

There are some limitations that should be considered. First, this study focused on the obesity condition, and the fecal microbiota of the rats with NCD were not detected. Second, the use of 16S rRNA gene sequencing rather than metagenomic sequencing limited the detection of bacterial taxonomy and functions. Nonetheless, we preliminary observed the changes in compositions and predicted functions caused by sucralose and sucrose. The different strains and the potential mechanisms should be further explored *in vitro* and *in vivo*. Finally, given glucose homeostasis was maintained by multiple organs, the weak connection of biochemical variables and fecal microbiota is also a limitation of this study.

In conclusion, our study demonstrated that 4-week dosages of sucralose (0.54 and 0.78 mM) changed the compositions of fecal microbiota in HFD-induced obese rats. Lower doses of sucralose (0.54 and 0.78 mM) tended to reduce the beneficial bacteria, *Lactobacillaceae* and *Akkermansiaceae*. Furthermore, 0.54 mM sucralose increased the predictive functions of carbohydrates and the consumption of 0.78 mM sucralose was related to amino acid metabolism. The effects of sucralose on energy metabolism might vary with dosages and intervention period. The metabolic effects of sucralose in different dosages should be considered in the future study.

## Data Availability Statement

The datasets presented in this study can be found in online repositories. The names of the repository/repositories and accession number(s) can be found below: NCBI [accession: PRJNA773931].

## Ethics Statement

The animal study was reviewed and approved by Ethics Committee of Renji Hospital Affiliated to Shanghai Jiaotong University.

## Author Contributions

MZ did the data analysis and prepared the manuscript. JC wrote the manuscript. MiY collected the samples and did the fecal DNA extraction. CQ, YL, YQ, and RF performed the animal experiments. MeY checked the data analysis. WL contributed to the study design. JM was the guarantor of this study to ensure the accuracy and integrity of the data. All authors contributed to the article and approved the submitted version.

## Funding

This study was supported by the Shanghai Pujiang Program (2019PJD027), Shanghai Medicine and Health Development Foundation (SHMHDF, DMRFP_I_06), Shanghai Municipal Education Commission—Gaofeng Clinical Medicine Grant Support (20181807), 2019 management and construction project of hospital (CHDI-2019-A-01), and the National Natural Science Foundation of China (81800747).

## Conflict of Interest

The authors declare that the research was conducted in the absence of any commercial or financial relationships that could be construed as a potential conflict of interest.

## Publisher's Note

All claims expressed in this article are solely those of the authors and do not necessarily represent those of their affiliated organizations, or those of the publisher, the editors and the reviewers. Any product that may be evaluated in this article, or claim that may be made by its manufacturer, is not guaranteed or endorsed by the publisher.

## Supplementary Material

Supplementary Figure S1 PCoA. **(A)** Weighted unifrac. **(B)** Weighted unifrac. Each sample was represented by a dot (*n* = 6). CON, control group; N054, 0.54 mM sucralose; N078, 0.78 mM sucralose; S324, 324 mM sucrose.

Supplementary Figure S2 Ratio of the domain phyla and body weight of rats. **(A)** Ratio of *Firmicutes* to *Bacteroidetes*. **(B)** Ratio of *Bacteroidetes* to *Proteobacteria*. Kruskal–Wallis rank sum test (*n* = 6). **(C)** Body weight before and after diet-induced obesity. NCD, normal chow diet; HFD, high-fat diet. **(D)** Body weight of rats during treatment period. Mean ± standard error of mean.

Supplementary Figure S3 Correlation heatmap of fecal microbiota with biochemical variables. Spearman correlation analysis between the top 30 most abundant bacterial families and biochemical variables related to glucose homeostasis. GLP-1, glucagon-like peptide-1; GIP, gastric inhibitory peptide; AUC, area under curve during intragastric glucose tolerance test; HOMA-IR, homeostatic model assessment for insulin resistance (HOMA-IR). ^*^*p* < 0.05.

Supplementary Figure S4 Network analysis of the top 50 abundant families in 0.54 mM sucralose group. Spearman's correlation analysis was used and a connection between two nodes stands for significant (*p* ≥ 0.5 and *p* < 0.5). The red color means positive correlation and green means negative correlation.

Supplementary Table S1 Alpha diversity of fecal microbiota in four groups.

## Abbreviations

ABC: ATP-binding cassette
Ace-K: Acesulfame potassium
ADI: Acceptable daily intake
ANOVA: Analysis of variance
AS: Artificial sweeteners
ATP: Adenosinetriphosphate
COMP: Component
CON: Control
DNA: Deoxyribonucleic acid
FDA: Food and Drug Administration
HFD: High-fat diet
KEGG: Kyoto Encyclopedia of Genes and Genomes
LDA: Linear discriminant analysis
LEfSe: Linear discriminant analysis effect size
OTU: Operational taxonomic units
PCoA: Principal coordinates analysis
PCR: Polymerase chain reaction
PERMANOVA: Permutational multivariate ANOVA
PICRUSt: Phylogenetic Investigation of Communities by Reconstruction of Unobserved States
PLS-DA: Partial least squares-discriminant analysis
RNA: Ribonucleic acid
SCFAs: Short-chain fatty acids
SD: Sprague Dawley
SPF: Specific pathogen-free.

## References

[1] Calle EE Kaaks R. Overweight, obesity and cancer: epidemiological evidence and proposed mechanisms. Nat Rev Cancer. (2004) 4:579–91. 10.1038/nrc1408 15286738

[2] Malik VS Popkin BM Bray GA Despres JP Hu FB. Sugar-sweetened beverages, obesity, type 2 diabetes mellitus, and cardiovascular disease risk. Circulation. (2010) 121:1356–64. 10.1161/CIRCULATIONAHA.109.876185 20308626 PMC2862465

[3] Vos MB Kaar JL Welsh JA Van Horn LV Feig DI Anderson CAM. Added sugars and cardiovascular disease risk in children: a scientific statement from the American Heart Association. Circulation. (2017) 135:e1017–34. 10.1161/CIR.0000000000000439 27550974 PMC5365373

[4] Rother KI Conway EM Sylvetsky AC. How non-nutritive sweeteners influence hormones and health. Trends Endocrinol Metab. (2018) 29:455–67. 10.1016/j.tem.2018.04.010 29859661

[5] Johnson RK Lichtenstein AH Anderson CAM Carson JA Després JP Hu FB. Low-calorie sweetened beverages and cardiometabolic health: a science advisory from the American Heart Association. Circulation. (2018) 138:e126–40. 10.1161/CIR.0000000000000569 30354445

[6] Imamura F O'Connor L Ye Z Mursu J Hayashino Y Bhupathiraju SN. Consumption of sugar sweetened beverages, artificially sweetened beverages, and fruit juice and incidence of type 2 diabetes: systematic review, meta-analysis, and estimation of population attributable fraction. BMJ. (2015) 351:h3576. 10.1136/bmj.h3576 26199070 PMC4510779

[7] Swithers SE. Artificial sweeteners produce the counterintuitive effect of inducing metabolic derangements. Trends Endocrinol Metab. (2013) 24:431–41. 10.1016/j.tem.2013.05.005 23850261 PMC3772345

[8] Gardener H Elkind MSV. Artificial sweeteners, real risks. Stroke. (2019) 50:549–51. 10.1161/STROKEAHA.119.024456 30760171 PMC6389377

[9] Greenhill C. Gut microbiota: not so sweet–artificial sweeteners can cause glucose intolerance by affecting the gut microbiota. Nat Rev Endocrinol. (2014) 10:637. 10.1038/nrendo.2014.167 25246083

[10] Valdes AM Walter J Segal E Spector TD. Role of the gut microbiota in nutrition and health. BMJ. (2018) 361:k2179. 10.1136/bmj.k2179 29899036 PMC6000740

[11] Suez J Korem T Zeevi D Zilberman-Schapira G Thaiss CA Maza O. Artificial sweeteners induce glucose intolerance by altering the gut microbiota. Nature. (2014) 514:181–6. 10.1038/nature13793 25231862

[12] Abou-Donia MB El-Masry EM Abdel-Rahman AA McLendon RE Schiffman SS. Splenda alters gut microflora and increases intestinal p-glycoprotein and cytochrome p-450 in male rats. J Toxicol Environ Health Part A. (2008) 71:1415–29. 10.1080/15287390802328630 18800291

[13] AlDeeb OA Mahgoub H Foda NH. Sucralose. Profiles Drug Subst Excip Relat Methodol. (2013) 38:423–62. 10.1016/B978-0-12-407691-4.00010-1 23668410

[14] Ma J Bellon M Wishart JM Young R Blackshaw LA Jones KL. Effect of the artificial sweetener, sucralose, on gastric emptying and incretin hormone release in healthy subjects. Am J Physiol Gastrointest Liver Physiol. (2009) 296:G735–9. 10.1152/ajpgi.90708.2008 19221011 PMC2670679

[15] Mezitis NH Maggio CA Koch P Quddoos A Allison DB Pi-Sunyer FX. Glycemic effect of a single high oral dose of the novel sweetener sucralose in patients with diabetes. Diab Care. (1996) 19:1004–5. 10.2337/diacare.19.9.1004 8875098

[16] Wang QP Browman D Herzog H Neely GG. Non-nutritive sweeteners possess a bacteriostatic effect and alter gut microbiota in mice. PLoS ONE. (2018) 13:e0199080. 10.1371/journal.pone.0199080 29975731 PMC6033410

[17] Qian C Qi Y Feng R Yang M Zhang M Liu W. Sucralose can improve glucose tolerance and upregulate expression of sweet taste receptors and glucose transporters in an obese rat model. Eur J Nutr. (2020) 60:1809–17. 10.1007/s00394-020-02375-1 32860125

[18] Reagan-Shaw S Nihal M Ahmad N. Dose translation from animal to human studies revisited. FASEB J. (2008) 22:659–61. 10.1096/fj.07-9574LSF 17942826

[19] Zhang M Feng R Yang M Qian C Wang Z Liu W. Effects of metformin, acarbose, and sitagliptin monotherapy on gut microbiota in Zucker diabetic fatty rats. BMJ Open Diab Res Care. (2019) 7:e000717. 10.1136/bmjdrc-2019-000717 31641523 PMC6777410

[20] Tremaroli V Bäckhed F. Functional interactions between the gut microbiota and host metabolism. Nature. (2012) 489:242–9. 10.1038/nature11552 22972297

[21] Chi L Bian X Gao B Tu P Lai Y Ru H. Effects of the artificial sweetener neotame on the gut microbiome and fecal metabolites in mice. Molecules. (2018) 23:367. 10.3390/molecules23020367 29425148 PMC6017827

[22] Qin J Li R Raes J Arumugam M Burgdorf KS Manichanh C. A human gut microbial gene catalogue established by metagenomic sequencing. Nature. (2010) 464:59–65. 10.1038/nature08821 20203603 PMC3779803

[23] Thomson P Santibanez R Aguirre C Galgani JE Garrido D. Short-term impact of sucralose consumption on the metabolic response and gut microbiome of healthy adults. Br J Nutr. (2019) 122:856–62. 10.1017/S0007114519001570 31258108

[24] Uebanso T Ohnishi A Kitayama R Yoshimoto A Nakahashi M Shimohata T. Effects of low-dose non-caloric sweetener consumption on gut microbiota in mice. Nutrients. (2017) 9:560. 10.3390/nu9060560 28587159 PMC5490539

[25] Dalenberg JR Patel BP Denis R Veldhuizen MG Nakamura Y Vinke PC. Short-term consumption of sucralose with, but not without, carbohydrate impairs neural and metabolic sensitivity to sugar in humans. Cell Metab. (2020) 31:493–502.e7. 10.1016/j.cmet.2020.01.014 32130881 PMC7784207

[26] Rodriguez-Palacios A Harding A Menghini P Himmelman C Retuerto M Nickerson KP. The artificial sweetener splenda promotes gut proteobacteria, dysbiosis, and myeloperoxidase reactivity in Crohn's disease-like ileitis. Inflamm Bowel Dis. (2018) 24:1005–20. 10.1093/ibd/izy060 29554272 PMC5950546

[27] Rizzatti G Lopetuso LR Gibiino G Binda C Gasbarrini A. Proteobacteria: a common factor in human diseases. Biomed Res Int. (2017) 2017:9351507. 10.1155/2017/9351507 29230419 PMC5688358

[28] Bian X Chi L Gao B Tu P Ru H Lu K. The artificial sweetener acesulfame potassium affects the gut microbiome and body weight gain in CD-1 mice. PLoS ONE. (2017) 12:e0178426. 10.1371/journal.pone.0178426 28594855 PMC5464538

[29] Depommier C Everard A Druart C Plovier H Van Hul M Vieira-Silva S. Supplementation with Akkermansia muciniphila in overweight and obese human volunteers: a proof-of-concept exploratory study. Nat Med. (2019) 25:1096–103. 10.1038/s41591-019-0495-2 31263284 PMC6699990

[30] Bian X Chi L Gao B Tu P Ru H Lu K. Gut Microbiome response to sucralose and its potential role in inducing liver inflammation in mice. Front Physiol. (2017) 8:487. 10.3389/fphys.2017.00487 28790923 PMC5522834

[31] Vandeputte D Falony G Vieira-Silva S Tito RY Joossens M Raes J. Stool consistency is strongly associated with gut microbiota richness and composition, enterotypes and bacterial growth rates. Gut. (2016) 65:57–62. 10.1136/gutjnl-2015-309618 26069274 PMC4717365

[32] Mack I Cuntz U Grämer C Niedermaier S Pohl C Schwiertz A. Weight gain in anorexia nervosa does not ameliorate the faecal microbiota, branched chain fatty acid profiles, and gastrointestinal complaints. Sci Rep. (2016) 6:26752. 10.1038/srep26752 27229737 PMC4882621

[33] Cao W Chin Y Chen X Mi Y Xue C Wang Y. The role of gut microbiota in the resistance to obesity in mice fed a high fat diet. Int J Food Sci Nutr. (2020) 71:453–63. 10.1080/09637486.2019.1686608 31774018

[34] Waters JL Ley RE. The human gut bacteria Christensenellaceae are widespread, heritable, and associated with health. BMC Biol. (2019) 17:83. 10.1186/s12915-019-0699-4 31660948 PMC6819567

[35] Lecomte V Kaakoush NO Maloney CA Raipuria M Huinao KD Mitchell HM. Changes in gut microbiota in rats fed a high fat diet correlate with obesity-associated metabolic parameters. PLoS ONE. (2015) 10:e0126931. 10.1371/journal.pone.0126931 25992554 PMC4436290

[36] Wu F Guo X Zhang J Zhang M Ou Z Peng Y. Phascolarctobacterium faecium abundant colonization in human gastrointestinal tract. Exp Ther Med. (2017) 14:3122–6. 10.3892/etm.2017.4878 28912861 PMC5585883

[37] Canfora EE Meex RCR Venema K Blaak EE. Gut microbial metabolites in obesity, NAFLD and T2DM. Nat Rev Endocrinol. (2019) 15:261–73. 10.1038/s41574-019-0156-z 30670819

