# Supplementary figures and images for: Low Doses of Sucralose Alter Fecal Microbiota in High-Fat Diet-Induced Obese Rats
Source: Front Nutr. 2021 Dec 28;8:787055. doi: 10.3389/fnut.2021.787055 (PMC8751733; doi:10.3389/fnut.2021.787055)

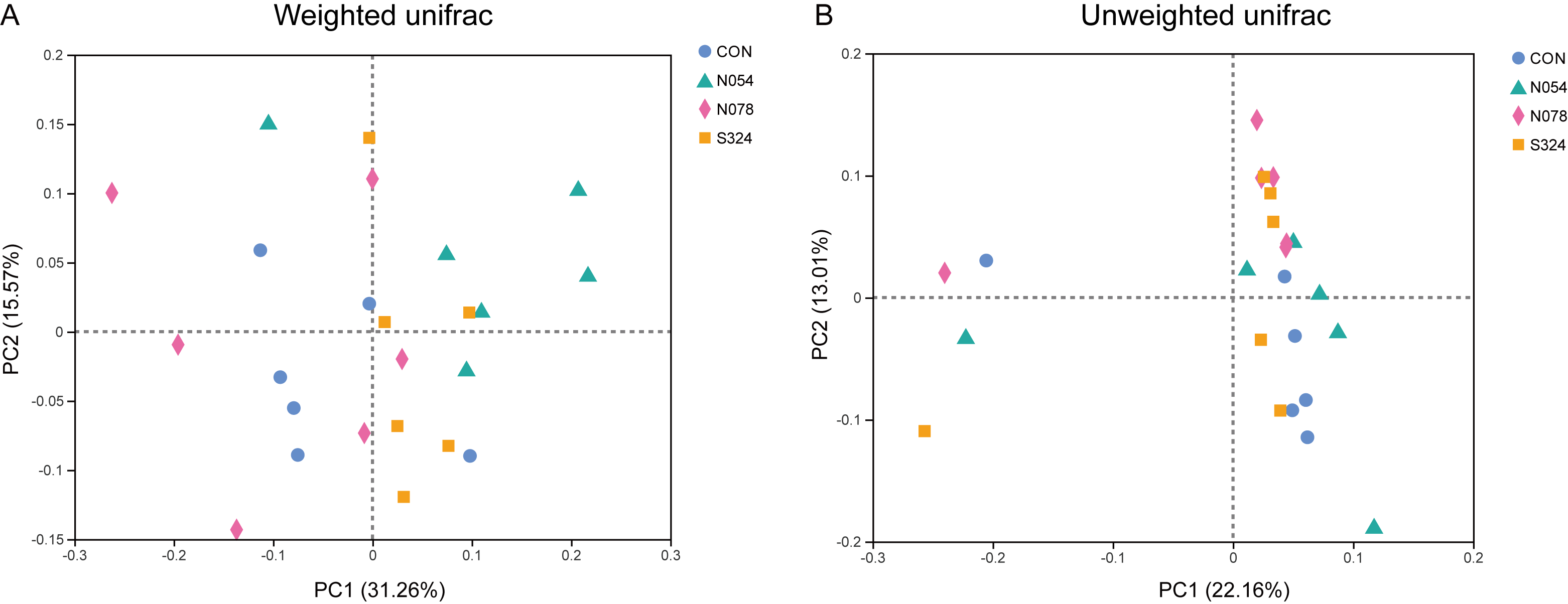

Supplement: Supplementary Figure S1 — PCoA. (A) Weighted unifrac. (B) Weighted unifrac. Each sample was represented by a dot (n = 6). CON, control group; N054, 0.54 mM sucralose; N078, 0.78 mM sucralose; S324, 324 mM sucrose. [file Image_1.TIF]

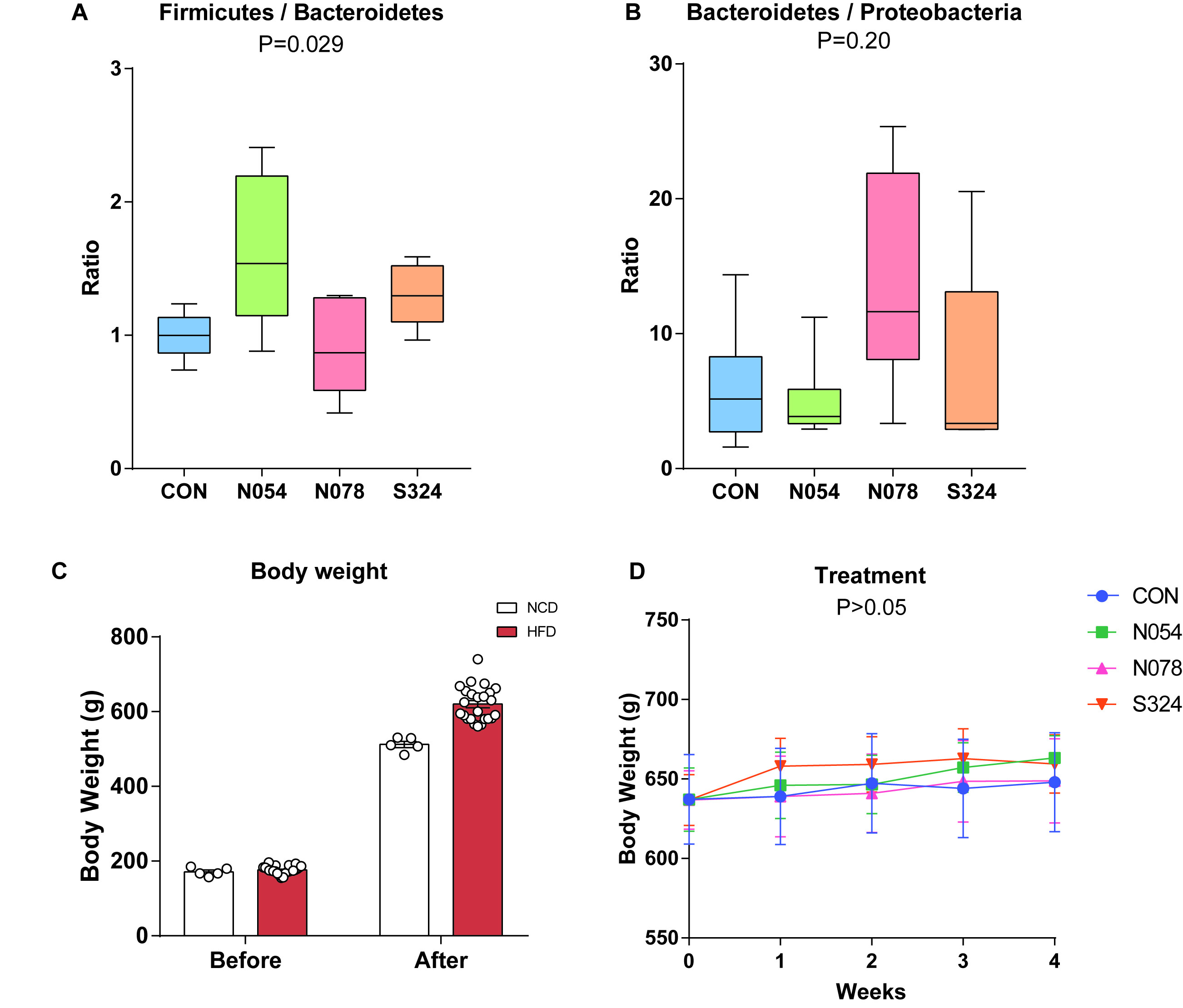

Supplement: Supplementary Figure S2 — Ratio of the domain phyla and body weight of rats. (A) Ratio of Firmicutes to Bacteroidetes. (B) Ratio of Bacteroidetes to Proteobacteria. Kruskal–Wallis rank sum test (n = 6). (C) Body weight before and after diet-induced obesity. NCD, normal chow diet; HFD, high-fat diet. (D) Body weight of rats during treatment period. Mean ± standard error of mean. [file Image_2.TIF]

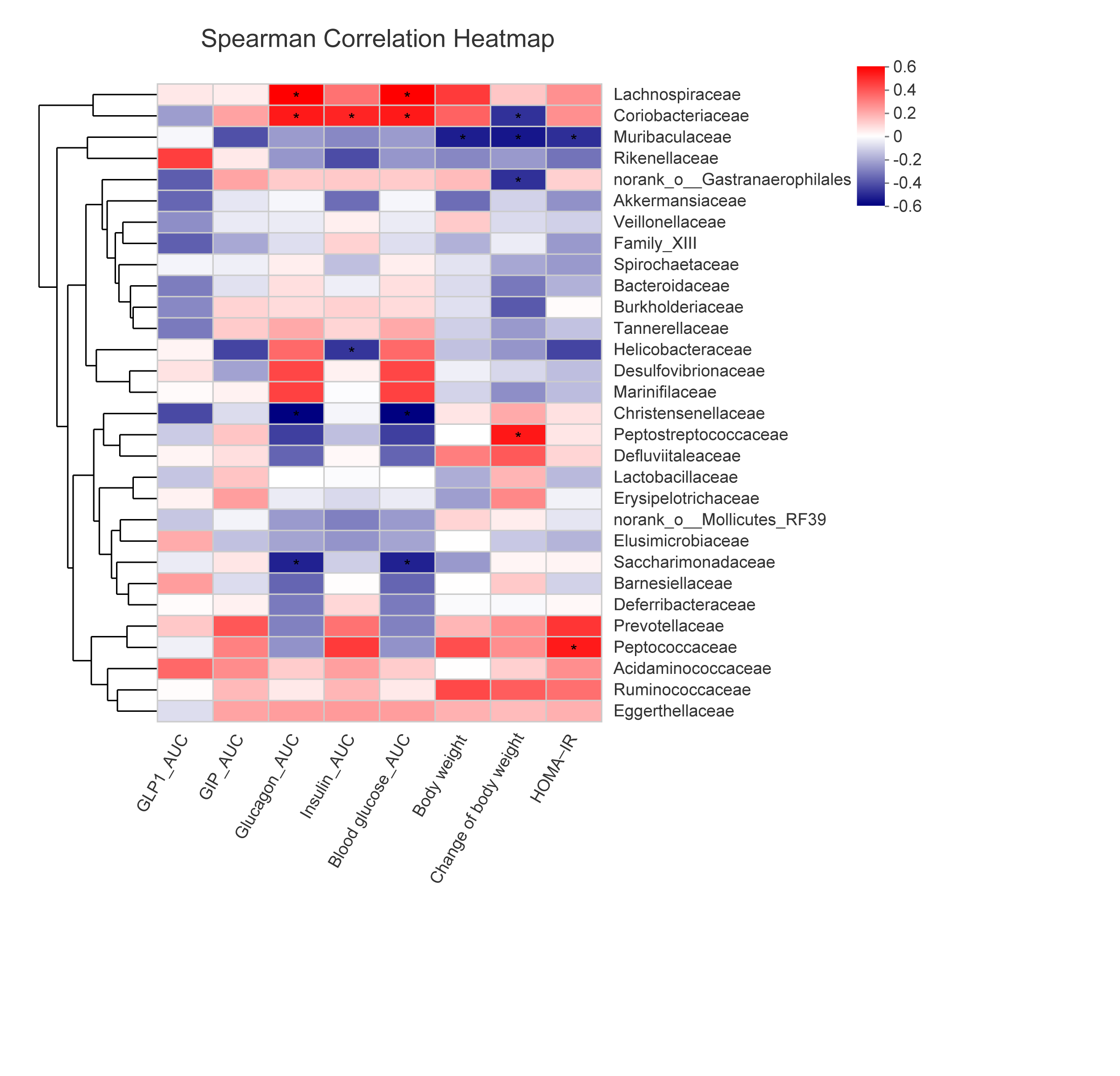

Supplement: Supplementary Figure S3 — Correlation heatmap of fecal microbiota with biochemical variables. Spearman correlation analysis between the top 30 most abundant bacterial families and biochemical variables related to glucose homeostasis. GLP-1, glucagon-like peptide-1; GIP, gastric inhibitory peptide; AUC, area under curve during intragastric glucose tolerance test; HOMA-IR, homeostatic model assessment for insulin resistance (HOMA-IR). *p < 0.05. [file Image_3.TIF]

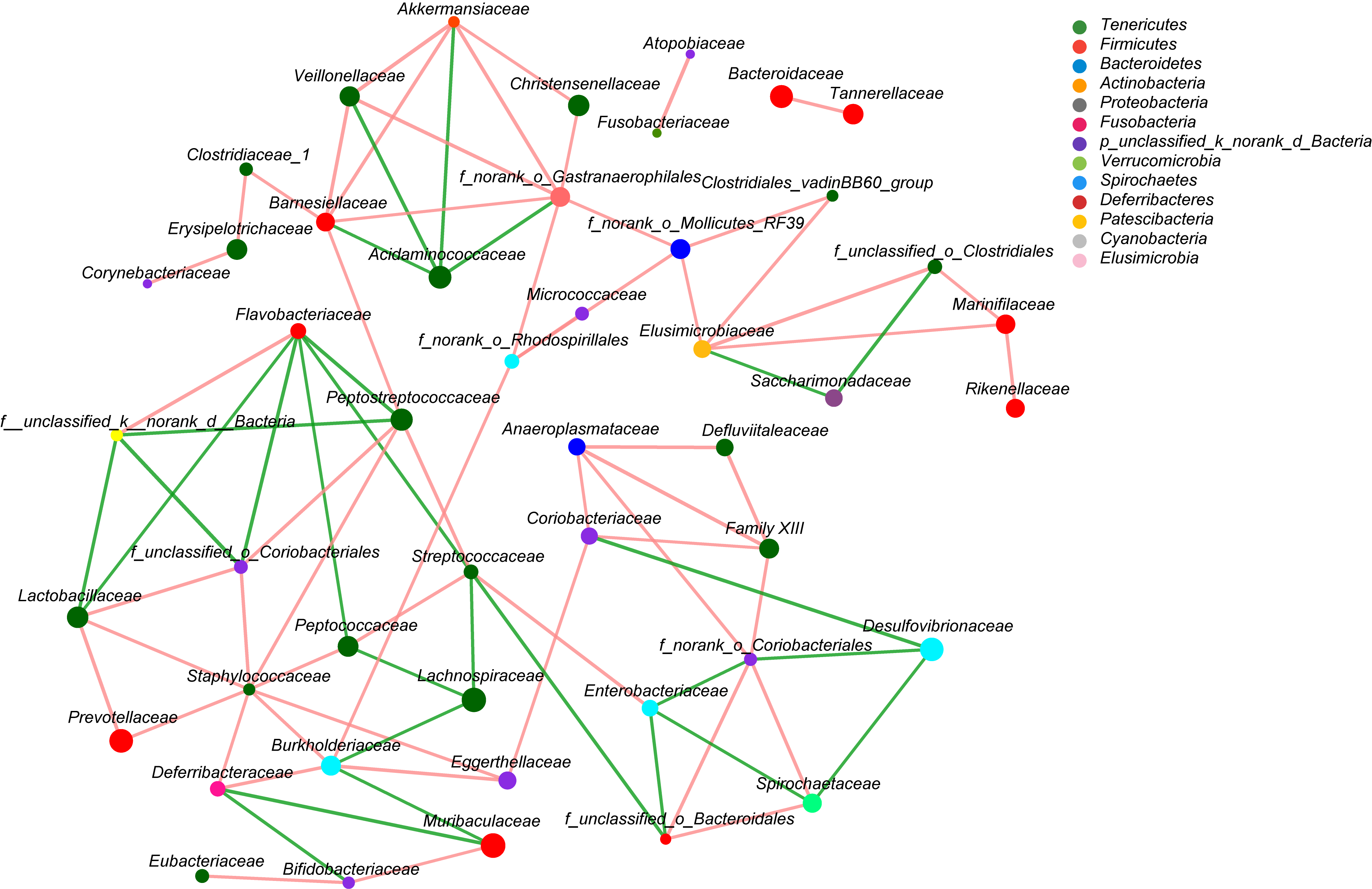

Supplement: Supplementary Figure S4 — Network analysis of the top 50 abundant families in 0.54 mM sucralose group. Spearman's correlation analysis was used and a connection between two nodes stands for significant (p ≥ 0.5 and p < 0.5). The red color means positive correlation and green means negative correlation. [file Image_4.TIF]
