# Supplementary material for: Low Doses of Sucralose Alter Fecal Microbiota in High-Fat Diet-Induced Obese Rats
Source: Front Nutr. 2021 Dec 28;8:787055. doi: 10.3389/fnut.2021.787055 (PMC8751733; doi:10.3389/fnut.2021.787055)
Supplement: Supplementary Table S1 — Alpha diversity of fecal microbiota in four groups. [file Table_1.DOCX]

**Supplementary**

**Table S1 Alpha diversity of fecal microbiota in four groups**

|  | CON | N054 | N078 | S324 |
| --- | --- | --- | --- | --- |
| Sobs | 450.83 ± 54.48 | 442.00±43.10 | 494.17±34.84 | 469.83±46.26 |
| ACE | 523.48±65.95 | 507.45±49.10 | 566.75±37.35 | 545.56±45.18 |
| Chao1 | 535.65±76.72 | 523.98±64.04 | 579.62±45.81 | 564.02±45.53 |
| Shannon | 4.25±0.22 | 4.31±0.23 | 4.32±0.29 | 4.38±0.24 |
| Simpson | 0.04±0.01 | 0.04±0.01 | 0.04±0.02 | 0.03±0.01 |

CON: control group; N054: 0.54 mM sucralose; N078: 0.78 mM sucralose; S324: 324 mM sucrose. Mean ± standard error.
